# The Role of Interleukin-8 in Lung Inflammation and Injury: Implications for the Management of COVID-19 and Hyperinflammatory Acute Respiratory Distress Syndrome

**DOI:** 10.3389/fphar.2021.808797

**Published:** 2022-01-12

**Authors:** Maria Candida Cesta, Mara Zippoli, Carolina Marsiglia, Elizabeth Marie Gavioli, Flavio Mantelli, Marcello Allegretti, Robert A. Balk

**Affiliations:** ^1^ Dompé Farmaceutici SpA, Via Campo di Pile, L’Aquila, Italy; ^2^ Dompé Farmaceutici SpA, Via Tommaso De Amicis, Napoli, Italy; ^3^ Dompé U.S. Inc., One Marina Park Drive, Boston, MA, United States; ^4^ Department of Medicine, Division of Pulmonary and Critical Care Medicine, Rush Medical College and Rush University Medical Center, Chicago, IL, United States

**Keywords:** cytokine storm, neutrophils, NETs, COVID-19, ARDS, Interleukin 8 (IL-8), CXCR1/2

## Abstract

Severe Acute Respiratory Syndrome Coronavirus—2 (SARS CoV-2) has resulted in the global spread of Coronavirus Disease 2019 (COVID-19) and an increase in complications including Acute Respiratory Distress Syndrome (ARDS). Due to the lack of therapeutic options for Acute Respiratory Distress Syndrome, recent attention has focused on differentiating hyper- and hypo-inflammatory phenotypes of ARDS to help define effective therapeutic strategies. Interleukin 8 (IL-8) is a pro-inflammatory cytokine that has a role in neutrophil activation and has been identified within the pathogenesis and progression of this disease. The aim of this review is to highlight the role of IL-8 as a biomarker and prognostic factor in modulating the hyperinflammatory response in ARDS. The crucial role of IL-8 in lung inflammation and disease pathogenesis might suggest IL-8 as a possible new therapeutic target to efficiently modulate the hyperinflammatory response in ARDS.

## Introduction

The recent world-wide pandemic of Coronavirus Disease 2019 (COVID-19) caused by the Severe Acute Respiratory Syndrome Coronavirus-2 (SARS-CoV-2) has resulted in a dramatic increase in patients with acute respiratory failure and Acute Respiratory Distress Syndrome (ARDS), both of which are associated with increased mortality, healthcare cost, and post recovery morbidity (Berlin et al., 2020; Tan et al., 2021). Despite decades of clinical investigation to define specific treatment of ARDS, to date the only treatment strategies that have been demonstrated to improve survival are lung protective ventilatory support and prone positioning for patients with moderately severe and severe ARDS (Guérin et al., 2013; Fan et al., 2017; Horie et al., 2020). Recent attention has been given on defining specific phenotypes of ARDS patients that would be expected to favourably respond to precision management strategies (Matthay et al., 2020). One such phenotypic stratification that has demonstrated potential to enrich a study population for successful intervention is the differentiation of hyper- and hypo-inflammatory ARDS (Calfee et al., 2018). The hyperinflammatory phenotype is associated with a higher expected mortality rate compared to the hypo-inflammatory phenotype and may be amenable to treatment with an anti-inflammatory strategy that is administered at the correct time and in the right amount (Calfee et al., 2018; Horie et al., 2020; Ware et al., 2020). To test this strategy, it will be necessary to identify individuals early with a hyperinflammatory phenotype, and the crucial role of chemokine markers such as Interleukin 8 (IL-8) in modulating the inflammatory response.

### ARDS and Lung Injury

Acute Respiratory Distress Syndrome (ARDS) is an acute respiratory condition characterized by hypoxemia and bilateral lung infiltrates without cardiac involvement that may rapidly progress into respiratory failure (Ranieri et al., 2012). Patients are often classified by the Berlin Criteria to mild, moderate, or severe ARDS severity based upon their Pa02/Fi02 levels, and positive end-expiratory pressure (PEEP) requirements (Ranieri et al., 2012). ARDS has been previously identified as the cause of 10% of ICU admissions with up to 23% of patients requiring mechanical ventilation (Bellani et al., 2016). Morality rates for patients with ARDS who are admitted to the ICU range from 35–45% with the probability of survival decreasing as the severity increases (Bellani et al., 2016). The most common causes or risk factors for the development of ARDS include pneumonia and non-pulmonary sepsis, along with others including aspiration, pancreatitis, drug overdoses, smoke inhalation, and blood transfusions (Meyer et al., 2021).

Patients with ARDS can be further classified into a hyperinflammatory phenotype based upon an influx of neutrophil activity that occurs after pulmonary insult leading to tissue injury (Spadaro et al., 2019). Neutrophils can elicit a hyperdriven immune response to danger signals, such as Neutrophil Extracellular Traps (NETs), networks of extracellular fibers, primarily composed of DNA from neutrophils, able to bind pathogens. NETs allow neutrophils to destroy extracellular pathogens while minimizing damage to the host cells, but are also known to damage host cells upon excessive activation (Nirmala and Lopus, 2020). Upon activation by interleukin-8 (IL-8), lipopolysaccharide (LPS) or pharmacological agents like phorbol myristate acetate (PMA), neutrophils release granule proteins and chromatin to form NETs (Brinkmann et al., 2004). Thereafter, NETs immobilize pathogens, limit their spread, and destroy them through antimicrobial protein production. A process called “NETosis” is the expulsion of NETs from individual neutrophils but does not affect their function to capture bacteria. Beyond this antimicrobial action, NETs can also contribute to the pathogenesis of disease, due to either excessive formation or impaired removal, which can both produce toxic events for the host (Papayannopoulos, 2018). Histopathological analysis of COVID-19 lungs reveals abnormal extracellular matrix remodeling, proliferation of epithelial cells and presence of NET degradation products detected in patient plasma that are known to correlate with lung distress and are predictors of COVID-19 severity and progression (Pannone et al., 2021). NETs induced by SARS-CoV-2 promote immunothrombosis, which subsequently contributes to lung cell death, resulting in massive neutrophil infiltration in the lungs as well as formation of NETs that are hypothesized to potentiate the development of ARDS (Middleton et al., 2020).

**FIGURE 1:**
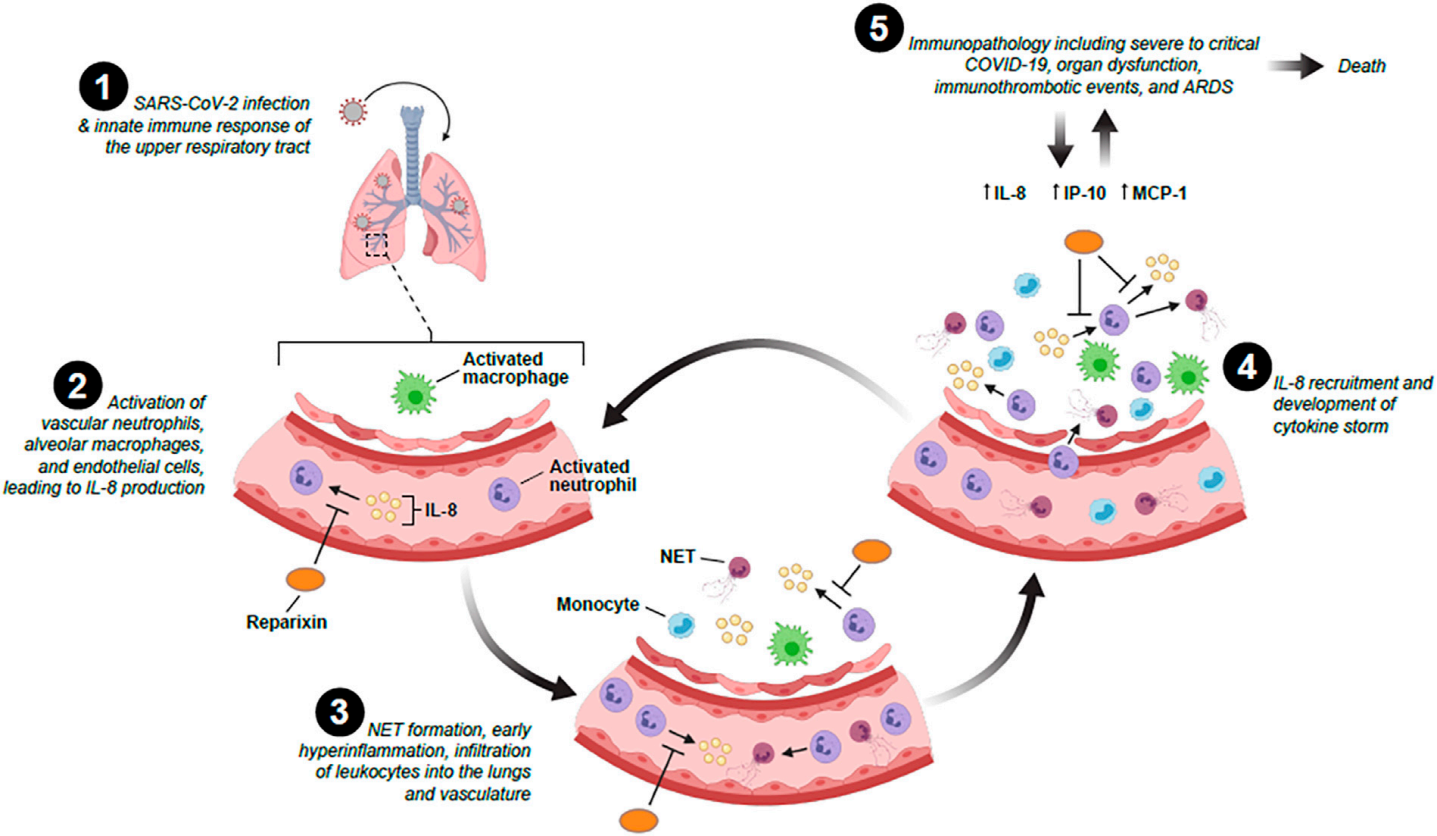
The potential role of IL-8 and IL-8 inhibitors in the recruitment, infiltration and activation of neutrophils following SARS-CoV-2 infection and cytokine storm development.

A hyperactive inflammatory response is a hallmark of COVID-19, with pulmonary and systemic inflammation common to most patients with severe COVID-19 (Del Valle et al., 2020). This is similar to past respiratory syndromes caused by Severe Acute Respiratory Syndrome Coronavirus (SARS-CoV) and Middle East Respiratory Syndrome Coronavirus (MERS-CoV) which were often associated with excessive production of pro-inflammatory cytokines, that led to pulmonary injury and ARDS (Peiris et al., 2003; Nassar et al., 2018). Patients affected by severe COVID-19 show a serum profile with significant increases of cytokines and chemokines (Huang et al., 2020; Blanco-Melo et al., 2020; Kox et al., 2020). An exaggerated inflammatory response to SARS-CoV-2 is associated with disease severity and mortality (Gustine and Jones, 2021; Fajgenbaum and June, 2020). SARS-CoV-2 entry and replication triggers an immune response, which can evolve into hyperactivation of the immune system associated with “cytokine storm” and cytokine release syndrome which are life threatening systemic inflammatory syndromes related to immune system dysregulation and uncontrolled over-production of soluble markers of inflammation (Fajgenbaum and June, 2020). The exuberant systemic inflammatory response of cytokine storm can lead to the development of ARDS and multiple organ system dysfunction/failure (Figure 1) (Fajgenbaum and June, 2020). “Cytokine storm” plays a key role in the pathophysiology of neutrophil influx from the circulation into highly vascularized organs, such as lungs and kidneys, that are the among targets in severe COVID-19 due to systemic inflammation (Le Stang et al., 2021).

### IL-8 and its Clinical Use

IL-8 is a potent neutrophil chemotactic factor that plays an important role under several pathological and physiological conditions. Numerous studies indicate that IL-8 is expressed in various cell types including neutrophils, fibroblasts, epithelial cells, hepatocytes, alveolar macrophages, and endothelial cells (Qazi et al., 2011). IL-8 mediates its biological effects through the binding to its cognate G-protein-coupled CXC chemokine receptors, CXCR1 and CXCR2, which activates a phosphorylation cascade to trigger chemotaxis and neutrophil activation as part of the inflammatory response (Holmes et al., 1991; Wu et al., 1996).

However, dysregulated signaling at the IL-8/CXCR1/2 axis has been identified as a possible cause to drive this immunopathology leading to an activated, prothrombotic, neutrophil phenotype characterized by degranulation and NET formation (Ha et al., 2017; Kaiser et al., 2021). By targeting IL-8/CXCR1/2, interference can occur within the cycle and attenuate neutrophil activation, degranulation, NETosis, and IL-8 release (Kaiser et al., 2021). IL-8 has been shown to have clinical use as a biomarker in diagnosing neonatal sepsis in which IL-8 levels are often produced after an infection by placental, monocytes, and endothelial cells. IL-8 has a mean sensitivity of 73%, and mean specificity of 81% when utilized to aid in diagnosing neonatal sepsis (Meem et al., 2011).

### IL-8 and Hyperinflammation ARDS

The crucial role of IL-8 in the pathogenesis of ARDS has been well known. Many authors propose the detection of IL-8 in the Bronchoalveolar lavage fluid (BALF) as a prognostic factor in patients at risk for non-COVID-19 ARDS, as well as predicting patient outcomes. BALF IL-8 concentrations have been significantly associated with mortality in sepsis, pneumonia, aspiration lung injury, transfusion-related acute lung injury (TRALI) of blood products, trauma related and non-specific ARDS (Kiehl et al., 1998; Kurdowska et al., 2001; Parsons et al., 2005; McClintock et al., 2008; Agrawal et al., 2012; Cartin-Ceba et al., 2015; Bime et al., 2019). Furthermore, IL-8 levels in BALF correlates negatively with arterial PaO2/FiO2 ratio. It has also been observed that trauma patients have neutrophil infiltration within their lungs associated with elevated IL-8 concentrations in BALF, supporting a possible role in organ injury (Pallister et al., 2002; Hildebrand et al., 2007). Additionally, patients with pancreatitis who developed ARDS have demonstrated significantly higher serum concentrations of IL-8, IL-6, and CD11b expression (indicative of neutrophil activation) compared to patients without ARDS (Browne and Pitchumoni, 2006).

In sepsis, patients at risk of ALI admitted to the ICU displayed serum levels of IL-8 related to the development of organ failure and half of them developed ARDS (Takala et al., 2002). Cigarette smoke exposure is associated with an increased risk of ARDS in smokers and non-smokers with and without lipopolysaccharide (LPS) inhalation; IL-8 plasma levels were found higher in patients who smoke (Moazed et al., 2016). In a study designed to explore the possible role of autophagy in ALI induced by seawater, it was found that lung injury was correlated with increased levels of IL-8 in BALF (Liu et al., 2013). In TRALI, elevated plasma levels of IL-8 preceded lung injury (Roubinian et al., 2015).

### IL-8 and the Pathophysiology of COVID-19

Chemokines are crucial mediators of inflammation that comprise an essential immune response needed to clear pathogens (Murdoch and Finn, 2000). However, in SARS-CoV-2 infection, infected monocytes and macrophages migrate to tissues and facilitate the spread of the virus (Gómez-Rial et al., 2020; Jafarzadeh et al., 2020). Several clinical studies report the infiltration of monocytes and macrophages into the lungs of COVID-19 patients contribute to the production of pro-inflammatory cytokines and chemokines that result in cytokine storm leading to tissue damage, organ system dysfunction and progression to ARDS as well as mortality (Costela-Ruiz et al., 2020; Gómez-Rial et al., 2020).

Patients with severe COVID-19 may progress to severe respiratory failure and/or ARDS, suggesting that immunopathology may drive the deleterious manifestations that are observed in the advanced stages of the disease (Gattinoni et al., 2020a; Gattinoni et al., 2020b). In fact, a clear compartmentalization of the T-cell lung population can be observed, with a peculiar leukocyte subpopulation pattern (depleted and exhausted CD4 and CD8 T-cell, higher fraction of T-reg cells in BALF) and a dominance of neutrophils, monocytes and macrophages characterized by a pronounced upregulation of surface markers related to activation (CD64, CD16, HLA-DR, CD11b, and CD69) (Ronit et al., 2021). Additionally, a wide range of cytokines are expressed at high levels in both the blood and in the lungs, notably IL-8, IP-10, and MCP-1. Among these, IL-8 exhibits a notable compartmentalized response within the lungs that, when considering the cellular immune response, is consistent with the well-established role of IL-8 in the recruitment of neutrophils to the lungs during acute pulmonary inflammation (Ronit et al., 2021).

Similar to patients with COVID-19, it is also known that during early pneumonia-related ARDS, bronchoalveolar NETs are associated with increased numbers of neutrophils and IL-8 concentration (Mikacenic et al., 2018). Several other studies have also demonstrated the potential contribution of NET formation in the inflammatory reaction, the immunopathology of COVID-19 ARDS, and the presence of NETs in the lungs of patients who died from COVID-19 (Barnes et al., 2020; Radermecker et al., 2020). Furthermore, in severe COVID-19 patients, immature and low-density neutrophils prevail with a greater propensity to release NETs, related to COVID-19 severity, which could explain a potential susceptibility towards progression of ARDS (Adrover et al., 2020). NET accumulation is also associated with pulmonary microvascular thrombosis, which triggers disease-related organ failure (Dolhnikoff et al., 2020; Leppkes et al., 2020).

BALF contains microenvironment information on bronchioles and lung alveoli. Its role in providing information about the pulmonary inflammation process could be crucial, and the association with plasma measurements could represent a strategic option to get a real perception of the inflammatory process within the lung compartment. Hyperinflammation of the lungs of severe COVID-19 patients is fueled by excessive production of chemokines. In fact, chemokines like CXCL1 (GRO*α*) and IL-8 were found to be 30 times more abundant in BALF than in plasma and 200 times more abundant than IL-6 and TNF-α; consistent with the levels of these chemotactic molecules, BALF was rich in neutrophils, lymphocytes and eosinophils (Bendib et al., 2021). A crucial aspect seems to be that plasma inflammatory cytokines/chemokines show limited correlations with BALF cytokines/chemokines, implying that circulating inflammatory molecules may not be a reliable proxy of the inflammation occurring in the lungs of severe COVID-19 patients (Zaid et al., 2021).

### Clinical Relevance of Elevated IL-8 Levels in COVID-19

The use of specific biomarkers in the management of COVID-19 patients may be useful to attenuate or prevent complications from the disease (Coperchini et al., 2020; Caruso et al., 2021). Much is already known of the role of IL-6 in COVID-19, and its involvement with the pathogenesis of cytokine storm, and disease severity. This has led to the repurposing of Tocilizumab, an anti-IL-6 receptor monoclonal antibody, in critical COVID-19 patients. However, there is a strong correlation of various other chemokines [IL-8, CXCL-10 (IP-10), CCL-2 (MCP-1), CCL3 (MIP-1a) and CCL-4 (MIP-1b)] with severity of illness in critical COVID-19, and others [IL-6, IL-8, TNF-α, IL-1β, IL-6, IL-8 and sTNFR1] have been demonstrated to be associated with severe COVID-19, including the presence of organ system failure (Del Valle et al., 2020; Li et al., 2020; McElvaney et al., 2020; Anderberg et al., 2021; Kaiser et al., 2021; Khalil et al., 2021; Meizlish et al., 2021). In patients with severe COVID-19, IL-8 is one of the main chemokines responsible for recruitment, activation, and accumulation of neutrophils. IL-8, was associated with the development of acute kidney injury, a complication of COVID-19, and respiratory failure as shown by a reduction in PaO_2_/FiO_2_ (Anderberg et al., 2021).

IL-8 has demonstrated to be significantly higher in non-survivors compared to survivors of COVID-19, and the dynamic change of the serum IL-8 levels has been correlated with the severity of the disease (Nagant et al., 2020; Li et al., 2021). Similarly, within 1,484 COVID-19 patients, IL-8 was associated with decreased survival even after controlling for covariates including patient demographics and comorbidities. Furthermore, within 663 COVID-19 patients, IL-8 levels were shown to be associated with worse survival after controlling for covariates including Sequential Organ Failure Assessment (SOFA) severity scale scores (HR: 1.6, *p* = 0.04) (Del Valle et al., 2020). IL-8 serum levels have also been shown to correlate better than IL-6 levels with overall clinical disease scores (Li et al., 2020; Nagant et al., 2020). Scoring cytokine storm by levels of MCP-3 and IL-8, accurately can stratify COVID-19 patients for high risk of mortality (Chen et al., 2020). Thus, supporting the possibility of using IL-8 as prognostic biomarker. It is important to note, that IL-8 levels may not always be elevated during a patient’s hospitalization stay, and it is hypothesized to peak during active infection at high viral loads and decrease thereafter as patients recover (Merza et al., 2021).

Elevated serum levels of IL-8 have been associated with longer duration of illness in patients with severe or critical COVID-19 (*p* = 0.004) (Ma et al., 2021). IL-8 has been associated with the recruitment and activation of polymorphonuclear-myeloid-derived suppressor cells (PMN-MDSC) which inhibit the response by T-cells to SARS-CoV-2 (Sacchi et al., 2020). Additionally, the frequency of PMN-MDSCs in critical COVID-19 patients is higher in non-survivors compared with survivors, and the frequency of PMN-MDSCs is positively correlated with plasma levels of IL-8 in hospitalized COVID patients (Sacchi et al., 2020). This suggests new mechanisms of cell regulation by a pivotal role for IL-8 signaling in the progression of the disease, and a potential therapeutic strategy for COVID-19 treatment.

### Novel Therapeutic Approaches Targeting the IL-8/CXCR1/CXCR2 Axis

Within studies in animal models of lung infection with influenza virus and *Streptococcus pneumoniae*, inhibitors of IL-8 receptors, such as CXCR1/2, showed a potential therapeutic benefit (Tavares et al., 2017). During both infections, a decreased morbidity was associated with decreased infiltration of neutrophils in the lungs, and a reduction of pulmonary damage and viral titers, without affecting bacteria burden. These data suggests that modulation of the inflammatory response by blocking CXCR1/2 improves disease outcome during respiratory influenza and pneumococcal infections, without compromising the ability of the murine host to deal with infection (Tavares et al., 2017).

Currently, two IL-8 inhibitors are under evaluation as potential therapeutic agents in patients with COVID-19. HuMax-IL-8 (BMS986253), is a human monoclonal antibody targeting IL-8 overexpressed in multiple cancer types, and able to reduce MDSCs (Dominguez et al., 2017). Reparixin, an allosteric inhibitor of IL-8 biological activity, is being investigated for its safety and efficacy in hospitalized adult patients with severe COVID-19 pneumonia, with results currently pending. The Phase three study was completed after promising results were noted in a phase two trial (NCT04794803, now NCT04878055) (REPAVID-19). Reparixin has also been found effective in significantly reducing neutrophil recruitment and accumulation to lung compartments, and improving gas exchange, in murine models of LPS-induced acute lung injury (ALI) (Zarbock et al., 2008). Additionally, recent evidence from healthy donors shows that IL-8 induces an increase in NET formation leading to granule release by neutrophils. This effect is reduced when incubating neutrophils with either an anti-IL-8 antibody or reparixin. A murine model of COVID-19 immunopathology blocking IL-8-like signaling with reparixin resulted in a trend towards clinical improvement of hACE2 mice at 24 h, reduced fibrinogen binding by intravascular neutrophils and attenuation of spike protein-induced pulmonary microthrombosis (Kaiser et al., 2021). Thus, this supports a useful preclinical proof of concept that neutrophil-IL-8-axis is a promising therapeutic target in treatment of severe COVID-19.

## Conclusion

Despite a multitude of investigational studies over the past 50 years and considerable advances in our understanding of the pathophysiology of ARDS, clinical management continues to be primarily supportive. The lack of specific effective targeted therapy has been further highlighted during this evolving COVID-19 pandemic, that has resulted in severe acute respiratory failure, and ARDS. Mortality and morbidity of this devastating clinical condition continues to remain high, underlining the need to find new effective therapies to reduce mortality. To help define effective therapeutic strategies it will likely be necessary to uncover specific phenotypes, such as the hyperinflammatory ARDS population, to target specific therapeutic strategies. While the exact role of IL-8 in COVID-19 and ARDS progression are still under investigation, there is agreement on the important role of IL-8 in the progress of disease and neutrophil activation. Targeting the IL-8/CXCR1/CXCR2 axis could allow the opportunity to not only identify new therapeutics for the treatment of COVID-19-related ARDS, but also to provide the new therapeutics to treat ARDS of any origin or cause with the aim to modulate the inflammatory response and its clinical consequences.
